# Wavelet-Driven Multi-Band Feature Fusion for RGB-T Salient Object Detection

**DOI:** 10.3390/s24248159

**Published:** 2024-12-20

**Authors:** Jianxun Zhao, Xin Wen, Yu He, Xiaowei Yang, Kechen Song

**Affiliations:** 1 School of Software Engineering, Shenyang University of Technology, Shenyang 110870, China; zjx@smail.sut.edu.cn (J.Z.); heyu_142616@sut.edu.cn (Y.H.); bestyxw@smail.sut.edu.cn (X.Y.); 2 School of Mechanical Engineering & Automation, Northeastern University, Shenyang 110819, China; songkc@me.neu.edu.cn

**Keywords:** RGB-T, salient object detection, cross-modal fusion, convolutional neural networks

## Abstract

RGB-T salient object detection (SOD) has received considerable attention in the field of computer vision. Although existing methods have achieved notable detection performance in certain scenarios, challenges remain. Many methods fail to fully utilize high-frequency and low-frequency features during information interaction among different scale features, limiting detection performance. To address this issue, we propose a method for RGB-T salient object detection that enhances performance through wavelet transform and channel-wise attention fusion. Through feature differentiation, we effectively extract spatial characteristics of the target, enhancing the detection capability for global context and fine-grained details. First, input features are passed through the channel-wise criss-cross module (CCM) for cross-modal information fusion, adaptively adjusting the importance of features to generate rich fusion information. Subsequently, the multi-scale fusion information is input into the feature selection wavelet transforme module (FSW), which selects beneficial low-frequency and high-frequency features to improve feature aggregation performance and achieves higher segmentation accuracy through long-distance connections. Extensive experiments demonstrate that our method outperforms 22 state-of-the-art methods.

## 1. Introduction

Saliency object detection has numerous applications in the field of computer vision, primarily detecting the most prominent objects in a scene. In image segmentation, saliency detection can prioritize salient regions, improving segmentation efficiency and accuracy [1,2]. In object detection, it helps algorithms quickly locate key areas, thereby enhancing detection speed and accuracy [3]. In the medical field, saliency object detection can assist doctors in quickly identifying abnormal regions in organs [4,5]. Additionally, saliency detection finds wide applications in robotics and assisted driving.

However, these methods are primarily designed for monocular RGB cameras and perform poorly in low-light, overexposed, or noisy environments. Even with image enhancement algorithms, the results are often unsatisfactory. With the advancement of deep learning and information technology, thermal images have been introduced into saliency object detection. These images are less sensitive to lighting, compensating for the limitations of RGB images in information representation. However, insufficient fusion can result in the loss of valuable information, while overly complex fusion strategies may introduce excessive noise, affecting detection results. Therefore, effectively integrating the two different modalities and selecting information beneficial to the task is the primary challenge in RGB-T salient object detection.

Traditional methods primarily depend on manually designed saliency features and prior knowledge. For example, ref. [6] proposed a multitask manifold ranking with cross-modality consistency algorithm, ref. [7] proposed a multi-scale manifold ranking algorithm, and ref. [8] introduced a low-rank tensor model to learn an optimized graph affinity matrix, which can effectively suppress noisy values in the features. Although traditional methods achieve good detection results in some simple scenarios, their reliance on ranking algorithms and a large amount of prior knowledge limits their detection capability in complex scenes.

Deep learning has shown outstanding performance in visual tasks and can overcome some detection challenges in complex scenes. In recent years, many deep learning-based methods have emerged in the RGB-T SOD field, significantly improving detection accuracy. For example, ref. [9] proposed a dual decoder architecture that introduces global context and fully fuses bimodal features through cascaded multi-interaction modules to suppress bimodal bias. Ref. [10] introduced a parallel symmetric network (PSNet), which enhances bimodal feature representation through a cascaded aggregation module and integrates adjacent layer saliency cues using a parallel symmetric fusion module (PSF). Ref. [11] designed a dedicated MS module to capture cross-modal and cross-scale complementary information from RGB and thermal images, and by utilizing cross-level complementary information from different fusion levels, it generates accurate saliency maps with clear boundaries. Ref. [12] proposed a method that regulates cross-modal interaction by obtaining global illumination scores and uses RGB features to supplement thermal semantics during the encoding phase while enhancing RGB localization with thermal features during the decoding phase. Ref. [13] adopted an early fusion strategy to extract complementary information through simple concatenation, addition, and multiplication operations before inputting into the network. Additionally, it designed an SGM module to progressively refine low-level features with high-level semantics. Ref. [14] proposed a secondary fusion network called the global contextually guided lightweight network, which employs secondary cross-modal integration to remove redundant information while fusing and propagating effective modal information.

Although the above methods have achieved good results, some issues still remain. Most methods focus only on global features without distinguishing between low-frequency and high-frequency information, which may lead to an inability to fully separate noise from valuable information. The downside of this approach is that noise in the high-frequency information may be excessively amplified, resulting in detection outcomes that are less stable and accurate.

**Figure 1:**
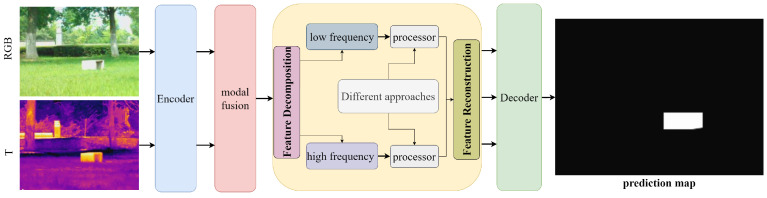
The design concept of our method.

Motivated by the aforementioned two challenges, we divide the features of the two modalities into channels, processing the information in different channels in parallel. We enhance the contextual information within the feature maps of each channel and establish global dependencies between feature maps to improve the perception of fine-grained features, thereby achieving thorough fusion. Our solution is illustrated in Figure 1. Additionally, by selectively utilizing low-frequency and high-frequency information, we aim to more accurately highlight salient information. The advantage of low-frequency information is that it retains more robust and stable global structural features, avoiding the noise interference caused by excessive attention to high-frequency details. Unlike [15,16,17], our method emphasizes low-frequency information during the fusion process, allowing us to capture the overall contours of salient objects more accurately. For high-frequency information, we use 3D convolution, enabling the network to better understand the relationships and contextual information between objects. This enhances the reliability and consistency of detection, thereby improving the model’s generalization capability and performance in handling complex scenes.

In summary, our contributions are as follows:

1. We propose a novel RGB-T SOD method, which consists of three components: a feature encoder, a channel-wise criss-cross Module, and a feature selection wavelet transformer.
2. We propose a channel-wise criss-cross module (CCM) that performs channel decomposition and parallel computation on features from both RGB and thermal modalities, effectively preventing information loss caused by direct fusion. This module employs attention mechanism to adaptively fuse complementary information from both modalities, and enhances the capture of global contextual information and fine-grained features through dynamic weight allocation, thereby achieving more comprehensive and robust feature fusion.
3. We propose a contextual feature selection wavelet transformer (FSW) module that uses wavelet transform to decompose fused feature information into high-frequency and low-frequency components. The high-frequency features capture fine-grained edge details for accurate target localization, while the low-frequency features provide background and structural context. This design enables the model to remain sensitive to object edges while leveraging global context to improve robustness, particularly in complex scenes. By effectively integrating both frequency bands during feature aggregation, the FSW module enhances segmentation accuracy and object localization.
4. Extensive experimental validation shows that our method achieves outstanding performance on three datasets.

## 2. Related Work

### 2.1. RGB Salient Object Detection

Siris et al. [18] proposed a context-aware learning method for salient object detection in complex scenes. Their approach includes a semantic scene context refinement and a context instance translator, which are used to enhance the contextual features learned from salient objects and to capture the relationship between objects and the scene context. Wang et al. [19] introduced the hybrid feature alignment network (HFANet) for salient object detection in optical remote sensing images (RSI-SOD), aiming to address issues such as complex backgrounds, multi-scale variations, and irregular edge topologies. They designed a hybrid encoder that combines the strengths of CNNs and transformers for local and global context modeling, respectively. Wu et al. [20] proposed a new method called dynamic pyramid convolution (DPConv), which dynamically selects the most appropriate kernel size according to the input image’s needs, enhancing feature representation at different scales. Wu et al. [21] also introduced the extreme downsampling network (EDN), which aims to enhance SOD performance by improving high-level features. They incorporated an extreme downsampling technique to effectively learn a global view, enabling precise salient object localization. Additionally, they designed a Scale-dependent pyramid convolution (SCPC) decoder to recover object details. Li et al. [22] proposed a lightweight framework using a Complementary Tri-decoder (CTD), which decouples the U-shaped structure into a semantic path, spatial path, and boundary path to address issues of semantic context dilution, spatial detail loss, and boundary refinement. The framework gradually optimizes segmentation results through a cross aggregation module (CAM) and a boundary refinement module (BRM), while the scale-adaptive pooling module (SAP) enhances the handling of multi-scale features.

### 2.2. RGB-D Salient Object Detection

Jin et al. [23] proposed the complementary depth network (CDNet), which selects depth maps containing saliency information as the training target and uses RGB features to estimate meaningful auxiliary depth maps, thus providing more saliency features. Chen et al. [24] introduced a novel model, RD3D, which for the first time attempts to use 3D convolutional neural networks to enhance cross-modal fusion capability. By employing pre-fusion in the encoder stage and deep fusion in the decoder stage, the model fully integrates RGB and depth information. Song et al. [25] proposed the modality-aware decoder (MaD), which focuses more on the relationships between modalities. Through modality-aware embedding and reasoning, MaD improves RGB-D fusion without the need for complex designs. Sun et al. [26] introduced the cascade aggregation transformer network (CATNet), which progressively enhances feature representation and fusion through multiple stages. The network also uses a cascade correction mechanism to address deficiencies in multi-scale feature fusion, thereby improving detection performance. Wu et al. [27] proposed the hierarchical depth-aware network (HiDAnet), which enhances the discriminative ability of RGB and depth features through a granularity-based attention mechanism. The network achieves coarse-to-fine multi-modal and multi-level fusion through a cross-dual attention module, while also introducing a multi-scale loss function to fully utilize hierarchical information. Zhang et al. [28] proposed a two-stage method: first, a generative network creates high-quality pseudo-depth images to calibrate the original depth data; then, cross-modal fusion is performed using a feature calibration and fusion network.

### 2.3. RGB-T Salient Object Detection

Gao et al. [29] proposed the multi-level multi-scale fusion network (MMNet), which explores the importance of feature response stages through self-attention, interactive attention, and adversarial combination, and integrates them into cross-modal features during the adversarial combination stage. Zhou et al. [14] utilized channel attention and spatial attention to fully fuse features of corresponding sizes from RGB and thermal modalities, enhancing the effectiveness and complementarity of feature representation. Tu et al. [9] introduced a multi-interactive dual decoder, which mines the complementarity of different modalities and the multi-type clues of image content, enabling the method to perform well even in the presence of invalid modalities. In another work, Tu et al. [30] designed a modality alignment module that combines spatial affine transformations, feature affine transformations, and dynamic convolutions to simulate the strong correlations between RGB and thermal (T) modalities. Song et al. [31] proposed a novel graph model-based weighted network, which differs from previous methods that directly fuse bimodal information at the convolutional feature layer. Instead, their method uses a graph model to more accurately represent the correlation between the two modalities. Wang et al. [32] introduced a T-aware early fusion network to improve the limitations of existing RGB-T datasets in extremely low-light scenarios. This network fully leverages the advantages of thermal images, using normal-light data during both training and testing, and validating its effectiveness with low-light and extremely low-light data. Zhou et al. [33] proposed the position-aware relational learning network (PRLNet), which addresses boundary blur and intra-class and inter-class variation issues by exploring pixel-wise distance and directional relationships through the signed distance map auxiliary module (SDMAM) and the feature refinement method with a direction field (FRDF).

## 3. Method

In this section, we will first provide an overview of our proposed network architecture. Then, in Section 3.2, we will detail our proposed CCM module, followed by the introduction of the FSW module in Section 3.2. Finally, in Section 3.4, we will discuss the loss functions used for network supervision.

### 3.1. Architecture Overview

**Figure 2:**
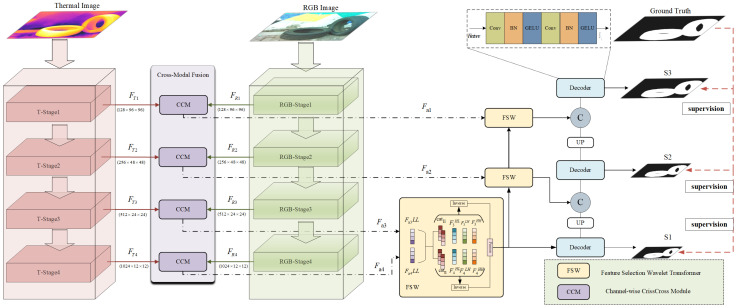
The overall architecture of our proposed method. Where UP represents upsampling.

Before the data are input into the network, the input data are first preprocessed, and the data from both modalities are cropped to the same size. The input to the network consists of two images of size (3 × 384 × 384), and the network output is a single-channel black-and-white image, where the black regions represent the background and the white regions represent the detected object, as shown in Figure 2. Benefiting from the excellent performance of the Swin Transformer in image tasks, we employ two Swin Transformer networks as the backbone networks. First, the images from the two modalities are fed into two separate backbone networks for feature encoding, resulting in multi-level features for both modalities: the feature information for the RGB image is denoted as $F_{R1},F_{R2},F_{R3},F_{R4}$, and the feature information for the thermal image is denoted as $F_{T1},F_{T2},F_{T3},F_{T4}$. Additionally, the number of channels for the four levels of features is 128,256,512,1024. Next, we design a cross-modal fusion module (CCM) to comprehensively facilitate information exchange between the two modalities, resulting in the fused information $F_{i}$. Subsequently, we design an FSW module based on the dual-tree complex wavelet transform, which fully utilizes the low-frequency and high-frequency information in the fused data to assist in object localization. Finally, the final saliency prediction map is obtained through a decoder.

### 3.2. Channel-Wise Criss-Cross Module

**Figure 3:**
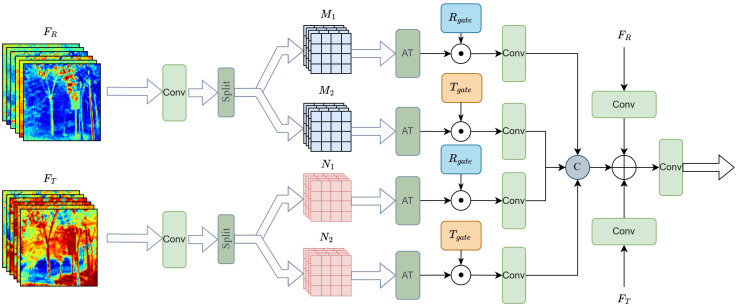
The detailed architecture of CCM.

The fusion of information from both modalities is a key step in RGB-T SOD tasks. Generally, RGB images capture rich detail information, while thermal images capture thermal characteristics of object surfaces. The two modalities complement each other, and in extreme environments, their interaction can compensate for the shortcomings of each, providing more enriched feature information for subsequent tasks. To fully leverage the feature information from both modalities and reduce redundant features, we propose a modality interaction method, as shown in Figure 3, to fuse the information from the two modalities. This module simultaneously focuses on both global and local features, enabling more thorough fusion of the two modalities’ information and utilizing the complementarity between the different modal features.

Compared to traditional fusion methods such as simple concatenation, early fusion, and late fusion, our proposed CCM offers several advantages. Simple concatenation directly combines features but lacks adaptive interaction between modalities. Early fusion may lose modality-specific information too early, while late fusion might miss important cross-modal correlations in the early stages. In contrast, our CCM enables both global and local feature interaction while preserving modality-specific characteristics through its unique structure.

First, we process the input features of the RGB and T modalities separately, using depthwise separable convolution (DW) to extract features at two different scales. The advantage of DW convolution is that it reduces the number of parameters and enhances computational efficiency while retaining the local information within each modality. This corresponds to the split operation shown in the figure. Two sets of features $M_{i}^{1}$, $M_{i}^{2}$ and $N_{i}^{1}$, $N_{i}^{2}$ are generated, respectively.

To better fuse the cross-modal information, we introduce the criss-cross attention mechanism [34]. Unlike simple fusion methods, this mechanism enables interactive computation of features along the spatial dimension, allowing the RGB and T modality features to focus on each other within local regions. Through the criss-cross attention mechanism, we independently process each set of features for the RGB and T modalities, obtaining features that are adaptively adjusted along the spatial dimension. These feature maps are enhanced within their respective modalities to capture local spatial dependencies. The specific process is as follows:

(1) $$M_{i}^{1},M_{i}^{2}=AT\left(Split\left(F_{i}^{R}\right)\right)$$

(2) $$N_{i}^{1},N_{i}^{2}=AT\left(Split\left(F_{i}^{T}\right)\right)$$

Here, $M_{i}^{1}$, $M_{i}^{2}$ and $N_{i}^{1},N_{i}^{2}$ represent the modality features enhanced by criss-cross attention, where AT denotes the criss-cross attention mechanism.

To adaptively adjust the importance of features at both global and fine-grained levels, we apply a global average pooling (GAP) operation to the original input features from the RGB and thermal T modalities, generating global feature representations. These representations are then processed through a Sigmoid activation function to produce weight maps, which effectively capture global contextual information and are used to regulate the relative importance of the modality-specific features. Subsequently, we further capture local feature details at the split feature level. This multi-scale feature processing strategy distinguishes our method from traditional early or late fusion approaches, as it maintains both global context and local details throughout the fusion process. We employ local convolution operations to conduct a more fine-grained analysis of different regions of the feature maps, ensuring that key salient target features are highlighted and details are better emphasized.

(3) $$R_{i}^{gate}=GAP\left(F_{i}^{R}\right)$$

(4) $$T_{i}^{gate}=GAP\left(F_{i}^{T}\right)$$

(5) $$F_{m}=Concat\left(R_{i}^{gate}⊙M_{i}^{1},R_{i}^{gate}⊙M_{i}^{2},T_{i}^{gate}⊙N_{i}^{1},T_{i}^{gate}⊙N_{i}^{2}\right)$$

$R_{gate}$ and $T_{gate}$ denote the weight maps for the RGB and T modalities, respectively. $F_{m}$ represents the fused features after applying the corresponding weights.

(6) $$F_{i}^{fuse}=F_{m}+Conv\left(F_{i}^{R}\right)+Conv\left(F_{i}^{T}\right)$$

Finally, we concatenate the four sets of weighted features along the channel dimension. Then, we perform element-wise addition of the original RGB features and T modality features with the weighted and fused features to form the final fused features $F_{i}^{fuse}$. This approach preserves important information from the original modalities while further enhancing the complementarity between the modalities, thus providing richer feature representations for salient object detection.

### 3.3. Feature Selection Wavelet Transformer

**Figure 4:**
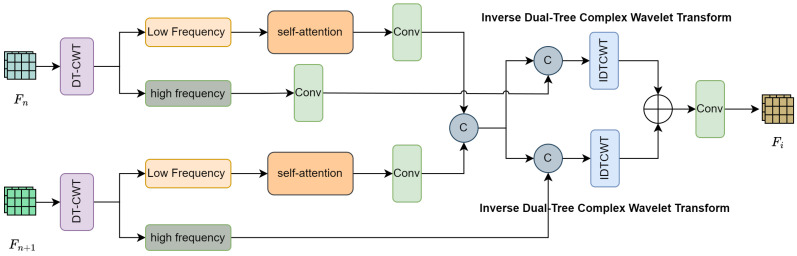
The structure of an FSW.

In general, the low-frequency information in an image contains the overall structure, such as gradients and large-scale components, while the high-frequency information contains more edge details. To more effectively utilize both the high-frequency and low-frequency information in images, we designed a feature selection wavelet transformer module, which aims to simultaneously focus on and filter the beneficial features in both frequency bands to improve the performance during the feature aggregation process. We specifically choose dual-tree complex wavelet transform (DT-CWT) [35] for its unique advantages in our task: (1) it provides approximate shift invariance, which helps maintain consistent feature representations; (2) it offers enhanced directional selectivity in high-frequency bands, which is crucial for capturing edge and texture information in RGB-T images; (3) it ensures perfect reconstruction with limited redundancy, making it computationally efficient while preserving important feature information. Moreover, wavelet transform is particularly suitable for RGB-T SOD tasks as it enables effective multi-scale feature decomposition and maintains complete spatial structure information during feature fusion, which is essential for accurate object localization. The frequency domain analysis it provides also helps in better capturing complementary information between RGB and thermal modalities, especially in challenging scenarios with poor illumination or complex backgrounds. The module’s design adopts long-range connections, directly linking the output of the previous layer to the next layer. These connections allow our method to better capture contextual information and achieve higher segmentation accuracy. The structure is shown in Figure 4.

The input features for the method are the fused features from two adjacent scales, namely $F_{i}^{fuse}$ and $F_{i+1}^{fuse}$. First, $F_{i+1}^{fuse}$ undergoes an upsampling operation to restore it to the same spatial dimensions as $F_{i}^{fuse}$. Subsequently, the features at adjacent scales are processed with the DT-CWT, which decomposes each feature into four sub-bands of different frequencies: a low-frequency sub-band (LL) and three high-frequency sub-bands (LH, HL, HH). Specifically, the LL sub-band captures the approximate coefficients and overall object structure, while the high-frequency sub-bands capture different directional details: HL for horizontal edges, LH for vertical edges, and HH for diagonal features. This allows for separate processing of high-frequency and low-frequency information. The specific process is as follows:

(7) $$\left({F_{n+1}}^{LL},{F_{n+1}}^{HL},{F_{n+1}}^{LH},{F_{n+1}}^{HH}\right)=DTCWT\left(Up\left(F_{n+1}^{fuse}\right)\right)$$

(8) $$\left({F_{n}}^{LL},{F_{n}}^{HL},{F_{n}}^{LH},{F_{n}}^{HH}\right)=DTCWT\left(F_{n}^{fuse}\right)$$

Here, $F_{n+1}$ and $F_{n}$ represent the feature information at adjacent levels, while DTCWT denotes the dual-tree complex wavelet transform operation.

After obtaining the low-frequency and high-frequency sub-bands, we apply different processing methods to these types of information to enhance the internal correlations between features. Given the different roles of these sub-bands, we prioritize their processing accordingly: Specifically, the low-frequency sub-band mainly contains global structural information, which is crucial for identifying the general location and rough boundaries of salient objects, thus receiving focused processing through a self-attention mechanism. The three high-frequency sub-bands (LH, HL, HH) collectively provide complementary directional information about edges and textures, and are therefore processed using 3D convolution operations, enabling the model to more effectively capture the global shape and local details of salient objects. Specifically, we transform the low-frequency sub-band features into query (Q), key (K), and value (V) matrices through linear mapping:

(9) $$Q=X_{LL}W_{Q},\;K=X_{LL}W_{K},\;V=X_{LL}W_{V}$$

(10) $$\mathrm{Attention}\left(Q,K,V\right)=\mathrm{softmax}\left(\frac{QK^{T}}{\sqrt{d_{k}}}\right)V$$

where $\left(W_{Q},W_{K},W_{V}\right)$ are the learned weight matrices, and the similarity between the Query and Key is calculated using the dot product, with $\frac{1}{\sqrt{d_{k}}}$ serving as a scaling factor to prevent excessively large values. The result is then normalized using the softmax function.

Subsequently, the low-frequency sub-bands of $F_{n}$ and $F_{n+1}$, enhanced by the self-attention mechanism, are concatenated to obtain the fused low-frequency feature representation, denoted as $cat_{ll}$. The remaining high-frequency sub-bands undergo convolutional processing, where 3D convolution is employed to handle the increased dimensionality of the high-frequency information. This results in the corresponding fused high-frequency features. The fused low-frequency features are then concatenated with the fused high-frequency features. An inverse transform using the inverse dual-tree complex wavelet transform (IDTCWT) is applied to reconstruct the fused features, thereby integrating information from different frequencies at a multi-scale level. Finally, the two resulting feature maps are combined element-wise to yield the final output of multi-scale fused features. We reconstruct the fused low-frequency and high-frequency features back to the original space using the inverse wavelet transform, producing two restored feature maps. These feature maps are subsequently added element-wise to generate the final output of multi-scale fused features.

(11) $$F_{r}=IDTCWT\left(cat_{ll},{F_{n}}^{HL},{F_{n}}^{LH},{F_{n}}^{HH}\right)$$

(12) $$F_{l}=IDTCWT\left(cat_{ll},{F_{n+1}}^{HL},{F_{n+1}}^{LH},{F_{n+1}}^{HH}\right)$$

(13) $$S=GeLU(BN\left(Conv\left(F_{r}+F_{l}\right)\right))$$

$cat_{ll}$ represents the concatenated low-frequency features, IDTCWT denotes the inverse wavelet transform, $F_{r}$ and $F_{l}$ represent the two aggregated sub-features, and *S* stands for the aggregated prediction value.

In summary, this module leverages multi-scale feature fusion in the frequency domain to not only preserve the global structure but also enhance fine-grained detection of salient targets, thereby improving overall performance.

### 3.4. Loss Function

In RGB-T salient object detection tasks, choosing an appropriate loss function is crucial for achieving accurate predictions. While several loss functions such as Focal Loss [36] and Dice Loss [37] are commonly used in segmentation tasks, we opt for a combination of binary cross entropy (BCE) [38] loss and intersection over union (IoU) [39] loss to supervise the network. This combination leverages the complementary advantages of both loss functions. The IoU loss primarily focuses on the spatial overlap between the prediction and the ground truth, while BCE loss emphasizes the predicted probability for each pixel. Compared to Focal Loss which mainly addresses class imbalance issues, our combination better handles the structural consistency requirements in RGB-T saliency detection. Additionally, unlike Dice Loss which may be sensitive to small objects and boundary regions, our approach provides better stability when dealing with thermal images where object boundaries might be less distinct.

(14) $${\mathrm{Loss}}_{BCE}=-\sum_{h=1}^{H}\sum_{w=1}^{W}\left(S\left(h,w\right)\cdot \log\left(\hat{S}\left(h,w\right)\right)+\left(1-S\left(h,w\right)\right)\cdot \log\left(1-\hat{S}\left(h,w\right)\right)\right)$$

$\hat{S}$ is the predicted saliency probability value at position (h,w), and *S* is the ground truth saliency label value at position (h,w). The variables *h* and *w* represent the height and width of the input image, respectively, while $Loss_{BCE}$ denotes the computed BCE loss value.

(15) $$\mathrm{IoU}=\frac{\mathrm{Intersection}}{\mathrm{Union}}=\frac{\sum_{h=1}^{H}\sum_{w=1}^{W}\left(\hat{S}\left(h,w\right)\cdot S\left(h,w\right)\right)}{\sum_{h=1}^{H}\sum_{w=1}^{W}\left(\hat{S}\left(h,w\right)+S\left(h,w\right)\right)-\sum_{h=1}^{H}\sum_{w=1}^{W}\left(\hat{S}\left(h,w\right)\cdot S\left(h,w\right)\right)}$$

(16) IoULoss=1−IoU

Intersection refers to the salient target areas that the model correctly predicts, while Union represents the combined area of the predicted saliency region and the ground truth saliency region. $\hat{S}\left(h,w\right)$ denotes the predicted probability of the salient target at position (h,w), and S(h,w) represents the label value at that position. The total loss value can be expressed as

(17) $$Loss_{all}=\sum_{i=1}^{3}\left(\mathrm{BCE}\left(\hat{S_{i}},S\right)+\mathrm{IoU}\left(\hat{S_{i}},S\right)\right)$$

$\hat{S_{i}}$ represents the predicted values at different stages of the network, and *S* denotes the ground truth map.

## 4. Experiments and Results

### 4.1. Datasets

**Figure 5:**
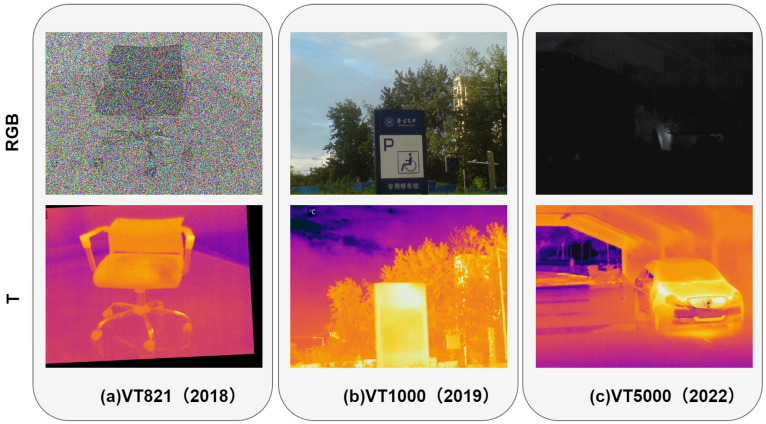
The dataset used in this experiment: (**a**) VT821, (**b**) VT1000, (**c**) VT5000.

To comprehensively evaluate our method, we conducted experiments using three RGB-T datasets: VT821 [6], VT1000 [40], and VT5000 [41]. The VT821 dataset consists of 821 manually registered RGB-T image pairs, which include several challenging single-modality failure cases. The VT1000 dataset contains 1000 well-registered image pairs, and the VT5000 dataset consists of 5000 pairs, featuring more complex scenes and higher levels of difficulty. For the experiments, we used 2500 image pairs from the VT5000 dataset as the training set, while the remaining data, along with the other two datasets, were used for testing. The sample data used for the three RGB-T datasets are shown in Figure 5.

### 4.2. Implementation Details

We implemented the proposed method using PyTorch 2.3.0 and CUDA 12.1, and conducted training and testing on a computer equipped with an NVIDIA RTX 3090 24 GB GPU. Following the standard settings of Swin Transformer, we resized all input images to 384 × 384. Data augmentation techniques such as flipping and cropping were applied to enhance model robustness. The Adam optimizer was used for training, with a batch size of 16 (determined through experiments testing values between 8 and 32) for 100 epochs. The initial learning rate was set to $1\times 10^{-4}$ (selected via grid search in the range [$1\times 10^{-5}$, $1\times 10^{-2}$]) for optimal convergence stability.

### 4.3. Evaluation Metrics

In this study, we used four evaluation metrics to assess the performance of our method: Structure-measure ($S_{m}$) [42], E-measure ($E_{m}$) [43], F-measure ($F_{m}$) [44], and mean absolute error (MAE) [45]. Specifically, $S_{m}$ is a spatial similarity metric used to evaluate the structural similarity between the predicted saliency map and the ground truth. $E_{m}$ is an enhanced alignment metric that considers both pixel-level and image-level statistical information. $F_{m}$ assesses the performance by combining precision and recall. MAE represents the average absolute error between the predicted values and the ground truth (GT), with smaller values indicating more accurate predictions. In addition to these four metrics, we also used the precision–recall (PR) curve and F-measure curve to evaluate the method’s performance.

### 4.4. Comparisons with State-of-the-Art Methods

#### 4.4.1. Quantitative Comparisons

We compared our method with 22 state-of-the-art approaches, including MTMR [6], M3S-NIR [7], MIDD [9], SGDL [40], ADF [41], TNet [12], MGAI [31], DMRA [46], EGNet [47], BASNet [48], PoolNet [49], R3Net [50], PFA [51], DCNet [30], CPD [52], S2MA [53], GRNet [54], SSOD [55], CGFNet [56], ACMANet [57], CAVER [58], and LSNet [59]. All experimental data were obtained from resources published by the respective authors.

**Figure 6:**
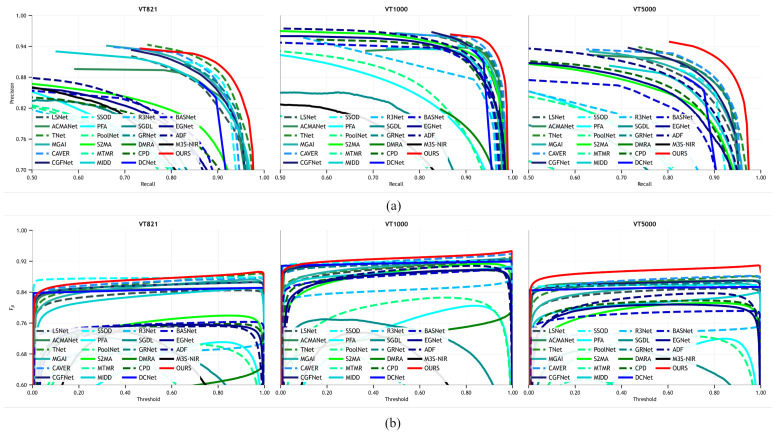
Quantitative comparison of our method with other state-of-the-art methods: (**a**) PR curve. (**b**) Fm curve.

As shown in Table 1, our method achieved excellent results across all three datasets. On the VT5000 dataset, our method reached an $S_{m}$ value of 0.917, an MAE of 0.024, an $F_{\beta }$ of 0.909, and an $E_{m}$ of 0.958. Compared to the second-best approach, our method improved $S_{m}$ by 1.88%, reduced the MAE by 1.42%, and increased $E_{m}$ by 1.48%. Our approach also demonstrated considerable improvements on the other two datasets. Traditional methods, such as MTMR and M3S-NIR, often rely heavily on prior knowledge, making it difficult for them to effectively represent features when combining the two modalities, resulting in unsatisfactory detection outcomes. RGB-based methods, without the complement of thermal information, tend to perform poorly in extreme environments. Additionally, as illustrated in Figure 6, the PR and F-measure curves further reflect our method’s performance across different datasets.

#### 4.4.2. Qualitative Comparison

**Figure 7:**
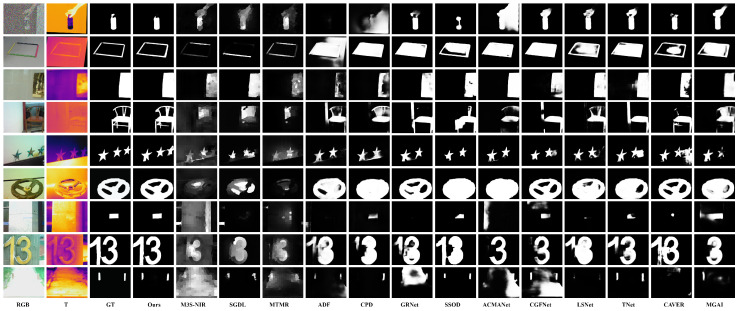
Qualitative comparison of our model with eleven recent state-of-the-art models.

Figure 7 presents a visual comparison between our method and several state-of-the-art approaches. The figure demonstrates that our method achieves more accurate detection results across various challenging scenarios, such as large targets, small targets, low contrast, overexposure, multiple targets, high noise levels, and target center offset. Compared to other methods, our approach consistently delivers better performance in these complex situations.

In the first row of Figure 7, which shows a noisy scene, with the advantage of our CCM module’s capability to adaptively fuse complementary information from RGB and thermal modalities, our method clearly detects the bottle’s shape with smooth edges and accurate detection while minimizing noise. In contrast, other methods often suffer from false positives and missed detections in this scenario, mainly due to their inability to effectively process information fusion between the two modalities. For example, while CAVER can detect the bottle, its result is somewhat distorted. Traditional methods like M3S-NIR and SGDL, due to their simple feature fusion strategies, produce incomplete contours, blurred shapes, and exhibit information loss.

In the second and sixth rows of Figure 7, leveraging the effective processing of high-frequency and low-frequency features by the FSW module, our method demonstrates superior performance in geometric shape detection. Specifically, the FSW module accurately captures target edge details through high-frequency components while maintaining structural integrity via low-frequency components, whereas other methods exhibit significant errors in edge and shape preservation. In the fourth row, where the target presents complex spatial structures, our approach, through the frequency decomposition mechanism of the FSW module, effectively preserves the intricate structural details and achieves precise edge localization, while other methods’ results show notable distortion. In the fifth row, with multiple salient targets positioned near image boundaries, the low-frequency features from the FSW module facilitate the preservation of global contextual information, resulting in more complete detection results, whereas most alternative methods produce incomplete results with higher noise levels. Finally, in the seventh and ninth rows, even under challenging conditions, such as RGB image overexposure or limited target feature information in thermal images, our method maintains accurate salient target extraction through the effective integration of different frequency components by the FSW module.

### 4.5. Ablation Study

**Figure 8:**
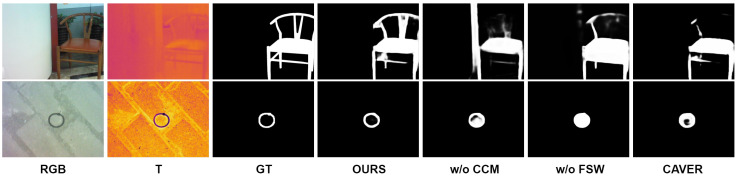
The visualization of ablation, where ’w/o’ stands for the absence of the corresponding module.

The experimental results demonstrate the necessity and effectiveness of multi-modal fusion in our approach. As shown in Table 2, by comparing three different modal combinations (RGB-RGB, T-T, and RGB-T), we observe that using complementary modalities (RGB-T) consistently outperforms using a single modality twice (RGB-RGB or T-T) across all evaluation metrics. This validates that the performance improvement comes from the effective fusion of complementary information between RGB and thermal modalities, rather than the complexity of network architecture. The results strongly support our design choice of leveraging both RGB and thermal inputs for more robust saliency detection. To confirm the impact of essential components in our model, we performed experiments where we either removed or substituted these elements from the complete configuration. The resulting quantitative data are presented in Table 3. Furthermore, to gain deeper insights into how these components contribute to performance, we included visualizations of the ablation study outcomes, displayed in Figure 8.

In this work, we incorporated three main modules: CCM, FSW, and DTCWT. To validate the contribution of each, we conducted experiments by either omitting or substituting them. The results of these experiments are detailed in Table 3. When the CCM module was replaced with direct addition, the model’s performance on all three datasets significantly declined. Without CCM, the MAE increased by 0.01 and $S_{m}$ decreased by 0.031 on VT5000. This is due to insufficient interaction between the two modalities, resulting in excessive noise in the fused information. The visualized ablation results reveal noticeable distortions and blurred edges when CCM is removed. When the FSW module was removed, the MAE increased by 0.013. The visualized results show that without FSW, the detection maps contain substantial noise, and much of the spatial structure of the detected objects is lost. This happens because removing the FSW module reduces the model’s attention to both global and fine-grained features, making it less effective in utilizing spatial features. We also validated the effectiveness of the network’s supervision approach. When using only BCE or IoU separately, the results were inferior compared to the combined supervision approach, indicating that joint supervision improves performance.

### 4.6. Failure Cases

**Figure 9:**
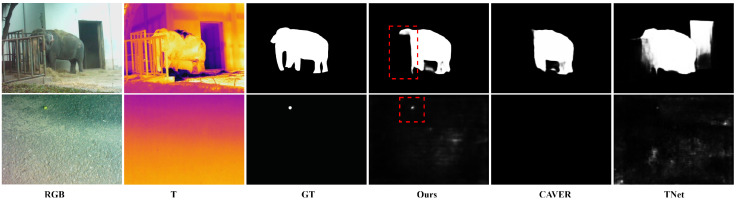
Visualization of some typical failure cases in our method.

Despite the strong performance of our method in various challenging scenarios, we identified some key failure cases that require further investigation. As shown in Figure 9 (first row), our method struggles to detect occluded parts when the target is occluded and its color is similar to the occluder in the RGB image. This limitation arises from the difficulty in distinguishing the features of the target and the occluder, especially when thermal information is also compromised. To address this challenge, future work could introduce temporal consistency analysis and develop occlusion-aware feature aggregation mechanisms. Additionally, as shown in Figure 9 (second row), performance degrades when the thermal image provides limited contrast due to similar temperatures, and the target is small in the RGB image. In these cases, accurate localization becomes particularly challenging. To enhance performance in such scenarios, we propose developing multi-scale feature enhancement modules and introducing adaptive fusion strategies that dynamically adjust modality weights based on scene conditions. To systematically address these limitations, we could design a local–global detection mechanism in the future to enhance feature discrimination in complex scenes and integrate context-aware fusion strategies for challenging scenarios.

## 5. Conclusions

In this work, we present a wavelet-driven multi-band feature fusion approach for RGB-T salient object detection. To achieve effective multi-modal fusion, we design a feature fusion module based on gating and attention mechanisms, which assigns weights to different modal components and realizes cross-modal fusion between RGB and thermal features through channel splitting. Furthermore, we introduce a feature selection wavelet transformer module to enhance fine-grained multi-scale feature representation and global context modeling capabilities while preserving complete spatial structure information of objects. The effectiveness of our proposed method is demonstrated through comprehensive experimental evaluations. 

## Figures and Tables

**Table 1:** Quantitative results of our method and 22 compared methods. The best two results are shown in red and green. ↑ indicates that higher values are better, while ↓ indicates that lower values are better.

Methods	VT821				VT1000				VT5000			
	$S_{m}\;↑$	MAE↓	$F_{\beta }^{\max}\;↑$	$E_{m}^{\max}\;↑$	$S_{m}\;↑$	MAE↓	$F_{\beta }^{\max}\;↑$	$E_{m}^{\max}\;↑$	$S_{m}\;↑$	MAE↓	$F_{\beta }^{\max}\;↑$	$E_{m}^{\max}\;↑$
M3S-NIR	0.723	0.140	0.738	0.837	0.726	0.145	0.735	0.828	0.652	0.168	0.596	0.760
MTMR	0.725	0.108	0.690	0.812	0.706	0.119	0.715	0.836	0.680	0.114	0.613	0.792
SGDL	0.765	0.085	0.735	0.840	0.787	0.090	0.770	0.859	0.751	0.089	0.695	0.829
S2MA	0.829	0.081	0.779	0.855	0.921	0.029	0.913	0.952	0.855	0.055	0.812	0.895
PFA	0.761	0.096	0.711	0.854	0.813	0.078	0.805	0.888	0.748	0.099	0.719	0.857
DMRA	0.666	0.216	0.661	0.766	0.784	0.124	0.798	0.863	0.659	0.184	0.601	0.758
LSNet	0.879	0.033	0.845	0.921	0.926	0.023	0.922	0.963	0.877	0.037	0.850	0.924
BASNet	0.823	0.067	0.763	0.858	0.909	0.030	0.901	0.944	0.839	0.054	0.791	0.884
ADF	0.810	0.077	0.752	0.839	0.910	0.034	0.908	0.950	0.864	0.048	0.837	0.911
CPD	0.818	0.079	0.758	0.862	0.907	0.031	0.897	0.947	0.855	0.046	0.818	0.905
DCNet	0.877	0.033	0.851	0.920	0.923	0.021	0.919	0.961	0.872	0.035	0.853	0.925
EGNet	0.830	0.063	0.756	0.857	0.910	0.033	0.898	0.945	0.853	0.050	0.808	0.893
MIDD	0.871	0.045	0.851	0.918	0.907	0.029	0.906	0.952	0.856	0.046	0.839	0.913
PoolNet	0.788	0.082	0.707	0.842	0.849	0.063	0.826	0.904	0.788	0.080	0.727	0.852
R3Net	0.782	0.081	0.711	0.819	0.886	0.037	0.876	0.939	0.812	0.059	0.753	0.863
MGAI	0.891	0.031	0.873	0.935	0.929	0.021	0.926	0.966	0.883	0.034	0.862	0.931
CGFNet	0.880	0.038	0.866	0.920	0.923	0.023	0.923	0.959	0.883	0.035	0.869	0.927
SSOD	0.895	0.027	0.878	0.942	0.925	0.020	0.922	0.964	0.877	0.033	0.859	0.933
ACMANet	0.883	0.035	0.851	0.926	0.927	0.021	0.923	0.964	0.887	0.033	0.871	0.936
TNet	0.899	0.030	0.888	0.938	0.929	0.021	0.930	0.966	0.895	0.033	0.881	0.937
GRNet	0.893	0.031	0.866	0.933	0.931	0.018	0.927	0.966	0.888	0.034	0.870	0.931
CAVER	0.898	0.026	0.877	0.934	0.938	0.016	0.939	0.973	0.900	0.028	0.882	0.944
OURS	**0.910**	**0.025**	**0.892**	**0.943**	**0.942**	**0.015**	**0.946**	**0.979**	**0.917**	**0.024**	**0.909**	**0.958**

**Table 2:** Ablation study on different modal combinations.

	VT821				VT1000				VT5000			
	$S_{m}\;↑$	MAE↓	$F_{\beta }^{\max}\;↑$	$E_{m}^{\max}\;↑$	$S_{m}\;↑$	MAE↓	$F_{\beta }^{\max}\;↑$	$E_{m}^{\max}\;↑$	$S_{m}\;↑$	MAE↓	$F_{\beta }^{\max}\;↑$	$E_{m}^{\max}\;↑$
OnlyRGB	0.885	0.034	0.845	0.912	0.939	0.016	0.939	0.974	0.903	0.027	0.890	0.947
OnlyT	0.846	0.043	0.814	0.907	0.908	0.027	0.903	0.956	0.871	0.038	0.844	0.927
OURS	0.910	0.025	0.892	0.943	0.942	0.015	0.946	0.979	0.917	0.024	0.909	0.958

**Table 3:** Ablation experiment results. w/o means without the module or supervision.

Setting Type	Configuration	VT821			VT1000			VT5000		
		$S_{m}\;↑$	MAE↓	$F_{\beta }^{\max}\;↑$	$S_{m}\;↑$	MAE↓	$F_{\beta }^{\max}\;↑$	$S_{m}\;↑$	MAE↓	$F_{\beta }^{\max}\;↑$
Module	w/o CCM	0.883	0.030	0.863	0.925	0.024	0.917	0.886	0.034	0.876
	w/o FSW	0.875	0.034	0.859	0.922	0.025	0.913	0.879	0.037	0.868
	w/o DT-CWT	0.905	0.028	0.885	0.935	0.018	0.945	0.910	0.027	0.897
	OURS	0.910	0.025	0.892	0.942	0.015	0.946	0.917	0.024	0.909
Loss	w/o BCE	0.907	0.027	0.889	0.941	0.016	0.944	0.911	0.027	0.906
	w/o IoU	0.908	0.026	0.891	0.939	0.016	0.942	0.910	0.026	0.899
	BCE+IoU	0.910	0.025	0.892	0.942	0.015	0.946	0.917	0.024	0.909

## Data Availability

The datasets used in this study are available at https://chenglongli.cn/code-dataset/ (accessed on 1 November 2024). The evaluation tool is available at https://github.com/lartpang/PySODEvalToolkit (accessed on 1 November 2024).
